# Basal cytokeratins in breast tumours among *BRCA1*, *BRCA2 *and mutation-negative breast cancer families

**DOI:** 10.1186/bcr1863

**Published:** 2008-02-14

**Authors:** Hannaleena Eerola, Mira Heinonen, Päivi Heikkilä, Outi Kilpivaara, Anitta Tamminen, Kristiina Aittomäki, Carl Blomqvist, Ari Ristimäki, Heli Nevanlinna

**Affiliations:** 1 Department of Oncology, Helsinki University Central Hospital, Haartmaninkatu, 00029 HUS, Helsinki Finland; 2 Department of Obstetrics and Gynecology, Helsinki University Central Hospital, Haartmaninkatu, 00029 HUS, Helsinki, Finland; 3 Department of Pathology/HUSLAB and Haartman Institute, Helsinki University Central Hospital, Haartmaninkatu,00029 HUS, Helsinki, Finland; 4 Genome-Scale Biology Program, Biomedicum Helsinki, University of Helsinki, Haartmaninkatu, 00014 Helsinki, Finland; 5 Department of Pathology, Helsinki University Central Hospital, Haartmaninkatu,00029 HUS, Helsinki, Finland; 6 Department of Clinical Genetics, Helsinki University Central Hospital, Haartmaninkatu,00029 HUS, Helsinki, Finland

## Abstract

**Introduction:**

Finding new immunohistochemical markers that are specific to hereditary breast cancer could help us to select candidates for *BRCA1*/*BRCA2 *mutation testing and to understand the biological pathways of tumour development.

**Methods:**

Using breast cancer tumour microarrays, immunohistochemical expression of cytokeratin (CK)-5/6, CK-14 and CK-17 was evaluated in breast tumours from *BRCA1 *families (*n *= 46), *BRCA2 *families (*n *= 40), non-*BRCA1*/*BRCA2 *families (*n *= 358) and familial breast cancer patients with one first-degree relative affected by breast or ovarian cancer (*n *= 270), as well as from patients with sporadic breast cancer (*n *= 364). Staining for CK-5/6, CK-14 and CK-17 was compared between these groups and correlated with other clinical and histological factors.

**Results:**

CK-5/6, CK-14 and CK-17 were detected mostly among oestrogen receptor (ER)-negative, progesterone receptor (PR)-negative and high-grade tumours. We found the highest percentages of samples positive for these CKs among ER-negative/HER2-negative tumours. In univariate analysis, CK-14 was significantly associated with tumours from *BRCA1 *(39%; *P *< 0.0005), *BRCA2 *(27%; *P *= 0.011), and non-*BRCA1*/*BRCA2 *(21%; *P *< 0.005) families, as compared with sporadic tumours (10%). However, in multivariate analysis, CKs were not found to be independently associated with *BRCA1 *or *BRCA2 *mutation status, and the most effective predictors of *BRCA1 *mutations were age at onset, HER2 status, and either ER or PR status.

**Conclusion:**

Although our study confirms that basal CKs can help to identify *BRCA1 *mutation carriers, this effect was weaker than previously suggested and CKs did not independently predict *BRCA1 *mutation either from sporadic or familial breast cancer cases. The most effective, independent predictors of *BRCA1 *mutations were age at onset, HER2 status, and either ER or PR status, as compared with sporadic or non-*BRCA1*/*BRCA2 *cancers.

## Introduction

Human breast cancers form a heterogeneous group of cancers. There are many subclassifications, and attempts have been made to identify differences between and characterize such subgroups. Earlier immunohistochemical studies, and recently gene expression studies as well, have been conducted to classify tumours [1]. One of the subgroups proposed is the so-called basal-like group of tumours, which are associated with breast cancers that express neither oestrogen receptor (ER) nor human epidermal growth factor receptor (HER)2, but express frequently 'basal' cytokeratins (CKs) such as CK-5/6, CK-14 and CK-17. These CKs are intermediate filaments that are found in the cells of the basal layer of normal breast ducts. Therefore, the term 'basal' is generally used, although the origin of breast tumour cells expressing these CKs is unknown. Moreover, the definition of basal phenotype in breast tumours is conflicting; for example, not all tumours classified as basal-like by microaray analysis express basal CKs [2]. In general, these tumours are associated with high grade and stage [3], but conflicting findings have been reported regarding the independent prognostic significance of the basal phenotype [4]. Although basal CK expression may predict early relapse among nonselected tumours, the clinical outcome of basal tumours has been found to be similar to that of nonbasal ER-negative tumours [5].

The general features of basal phenotype tumours are the same as those of tumours in *BRCA1 *mutation carriers [1]. In particular, expression of CK-5/6 [6,7] and CK-5/14 [8] has been associated with *BRCA1 *tumours. Moreover, Lakhani and colleagues [9] tested five basal markers (CK-14, CK-5/6, CK-17, epidermal growth factor and osteonectin), all of which were significantly associated with *BRCA1 *tumours as compared with unselected breast tumours.

In addition to the CKs, it has been clear for some time that *BRCA1*-associated tumours have distinct pathological features when compared with sporadic cancers. *BRCA1 *tumours have higher tumour grade, less ER and progesterone receptor (PR) expression, less HER2 expression and over-expression of p53, and a higher proportion of medullary than sporadic cancers [10]. Furthermore, they exhibit high Ki-67-index and over-express proteins such as cyclin-E, cyclin-A and cyclin-B_1 _[10]. The pathological phenotype of *BRCA2*-associated tumours is inconsistent, and in many cases no significant differences have been found between *BRCA2*-associated and sporadic cancers [11-13]. However, some studies have found *BRCA2*-associated tumours to be negative for HER2 and to over-express CHEK2 (checkpoint kinase 2) and RAD51 (homolog of Saccharomyces cerevisiae Rad51) [10]. The basal CK frequencies have been found to be similar in this group of tumours and sporadic ones [9].

There is much need for markers that could be used to distinguish patients and families who are likely to carry a *BRCA1*/*BRCA2 *germline mutation from mutation-negative families and from breast cancer patients in general. Such markers could facilitate targeting of expensive and time-consuming genetic testing to individuals who are most likely to carry those mutations. It is also necessary to learn more about the biological characteristics of these tumours in order to foster development of less radical prevention strategies than risk-reducing surgery and to identify possible targets for drug development. It has been suggested that CK-5/6, CK-14, P-cadherin and epidermal growth factor receptor may have utility as a first-line immunohistochemical test for the presence of germline *BRCA1 *mutation [14]. In this study, we evaluated expression of basal CKs among tumours from *BRCA1*, *BRCA2 *and non-*BRCA*1/*BRCA2 *families (families with several breast cancer patients without *BRCA1 *or *BRCA2 *mutations), and sporadic breast cancer patients. It is important to compare tumours of mutation carriers with tumours from families including several cancer cases, and not just to compared them with sporadic tumours, because this is the situation usually encountered in the clinical setting of genetic counselling.

## Materials and methods

### Breast cancer patients and tumours

A series of 884 consecutive, newly diagnosed breast cancer patients was recruited from the Department of Oncology (Helsinki University Central Hospital) during the period from 1997 to 1998 and during the year 2000, as described in detail by Syrjäkoski and coworkers [15] and Kilpivaara and colleagues [16]. Familial breast cancer patients (*n *= 546) were recruited from the Departments of Oncology and Clinical Genetics, as described by Eerola and coworkers [17]. This series includes breast cancer families with at least three first-degree or second-degree relatives with breast or ovarian cancer, as well as patients with one first-degree relative affected by breast or ovarian cancer, with no restriction on age. All of the breast cancer families and unselected patients were tested for *BRCA1 *and *BRCA2 *mutations by mutation analysis of the entire coding sequences and exon/intron boundaries of the genes using protein truncation test and denaturing gradient gel electrophoresis, or as previously described [18-20], except for 158 patients from families with two affected members, in whom *BRCA1 *and *BRCA2 *mutation status was not determined.

We collected all available paraffin blocks containing enough tumour tissue from primary breast cancers of unselected and familial breast cancer patients, and the most representative area of the tumour was punched to produce a breast cancer tissue microarray (TMA), including two to four cores (diameter 0.6 mm) from each original block, as partially described previously [21]. Altogether, the TMAs included 1,335 invasive breast cancer tumours; 921 of these were from familial patients (577 from families with three or more first-degree or second-degree relatives with breast or ovarian cancer, including the proband, and 344 from families with two affected first-degree relatives) and 414 from sporadic patients (patients without the above-defined family history of breast or ovarian cancer). Fifty-two of the tumours from familial patients were from patients of families positive for *BRCA1 *mutation and 56 tumours were from patients of families positive for *BRCA2 *mutation.

In this study we excluded lobular carcinomas as ambiguous, as well as tumours from noncarriers in the *BRCA1*/*BRCA2 *families and tumour from patients for whom no representative tumour tissue was available on the TMA arrays. Finally, the staining findings was obtained for a total of 1,078 tumours, which included 622 cancers from the unselected breast cancer patient series. Combined with the family material, CK expression was evaluated in the following: 46 cancers from *BRCA1 *families; 40 cancers from *BRCA2 *families; 358 cancers from non-*BRCA1*/*2 *families; 270 cancers from patients with one first-degree or second-degree relative affected by breast or ovarian cancer; and 364 cancers from patients with sporadic breast cancer (patients with no family history of breast cancer).

We studied haematoxylin and eosin stained sections of the original blocks for histological diagnosis and grading (by the same pathologist [PH]) Grading was performed using the Scarff-Bloom-Richardson method, as modified by Elston and Ellis [22]. All of the TMA slides were stained with antibodies to CK-5/6, CK-14 and CK-17. Briefly, 5 μm sections were cut from paraffin-embedded blocks, deparafinated in xylene and dehydrated in a series of graded alcohols. Sections that were immnunostained with antibodies against CK-5/6 (dilution 1:25; Dako, Glostrup, Denmark) and CK-14 (dilution 1:20; Novocastra, Newcastle upon Tyne, UK) were first pretreated in a microwave oven in Tris-EDTA (pH 9.0), incubated with the primary antibodies for 30 minutes and visualized using the Envision detection system (Dako) and LabVision autostainer (Thermo Fisher Scientific Inc., Fremont, CA, USA). Sections that were immunostained with antibodies against CK-17 (dilution 1:20; Vector Laboratories, Burlingame, CA, USA) were first pretreated in a microwave oven in 10 mmol/l citric acid (pH 6.0), incubated with primary antibodies for 30 minutes and visualized using (Renaissance) TSA-Biotin system NEL 700 kit (PerkinElmer, Massachusetts, USA). Specimens were considered negative if immunopositivity was found in 0% to 10% and positive if 11% to 100% of the cancer cells exhibited immunopositivity. A cut-off value of >10% results in clearly positive staining.

All the TMA slides were stained using routine methodology with p53 antibodies (1:300; Dako, Copenhagen, Denmark; described previously by Eerola and coworkers [21]). Samples were considered positive when 20% of the cancer cells stained positive. Amplification of the HER2 oncogene was determined by chromogenic *in situ *hybridization (as described by Tanner and coworkers [23]); none to five replications was considered negative, and more than six replications was considered positive. Information on ER and PR status was acquired from pathology reports. The staining methodology and evaluation of the results are applied routinely in breast cancer diagnostics and are previously described by Eerola and coworkers [21]. Samples were considered positive when 10% of the cancer cells stained positive. The information on stage was acquired from patient records.

### Statistical analysis

Statistical analysis was conducted using SPSS for Windows (SPSS Inc., Chigaco, IL, USA). We tested the differences in dichotomous variables by χ^2 ^test or Fisher's exact test, and multivariate analysis was conducted using logistic regression (backward conditional, *P *for stepwise removal 0.05). All *P *values are two sided.

### Ethics

The study was performed with informed consent from the patients as well as with approval from the Ethics Committee (E8) of the Helsinki University Central Hospital and from the Ministry of Social Affairs and Health in Finland.

## Results

Tumours expressing basal CKs (CK-5/6, CK-14 and CK-17) were more likely to be ER negative and PR negative, and to occur in younger breast cancer patients. CK-5/6 expression was also significantly associated with high grade and p53 positivity, and expression of CK-5/6 and CK-14 with HER2 negativity. CK14 was significantly associated with *BRCA1 *mutation but also with *BRCA2 *and non-*BRCA1*/*BRCA2 *groups of cancers, as compared with sporadic breast cancers (Table 1). We found all three CK markers to be highly significantly associated with the subgroup of tumours negative for both ER and HER2. Within this subgroup of cancers, CK-5/6 was positive in 33% of 167 cancers (*P *< 0.0005), CK-14 in 35% of 161 cancers (*P *< 0.0005), and CK-17 in 10% of 158 cancers (*P *= 0.001), as compared with 9%, 11% and 4%, respectively, among the tumours in which one or both of the markers (ER of HER2) was positive.

**Table 1:** Association of CK-5/6, CK-14 and CK-17 with clinicopathological features

	All	%	CK-5/6			CK-14			CK-17		
			
			Positive/all^a^	%	*P*	Positive/all^a^	%	*P*	Positive/all^a^	%	*P*
All	1078	100	129/982	13.1		151/955	15.8		41/931	4.4	
Oestrogen receptor											
Negative	266	26.0	67/241	27.8	<0.0005	63/236	26.7	<0.0005	20/236	8.5	0.001
Positive	757	74.0	56/692	8.1		78/671	11.6		21/650	3.2	
Progesterone receptor											
Negative	388	38.0	74/353	21.0	<0.0005	76/348	21.8	<0.0005	26/346	7.5	<0.001
Positive	633	62.0	49/578	8.5		65/557	11.7		15/538	2.8	
Grade											
I	231	21.6	17/194	8.8	<0.0005	32/193	16.6	0.097	3/185	1.6	0.021
II	453	42.5	43/420	10.2		52/403	12.9		15/391	3.8	
III	383	35.9	69/359	19.2		65/350	18.6		23/347	6.6	
T											
1	649	61.3	69/580	11.9	0.159	89/556	16.0	0.790	27/549	4.9	0.434
2 to 4	409	38.7	58/386	15.0		59/384	15.4		14/366	3.8	
N											
0	596	56.6	72/538	13.4	0.607	85/524	16.2	0.477	23/506	4.5	0.659
1	457	43.4	52/424	12.3		60/413	14.5		16/405	4.0	
M											
0	997	96.5	122/910	13.4	0.882	144/885	16.3	0.269	39/861	4.5	0.706
1	36	3.5	4/32	12.5		3/33	9.1		1/32	3.1	
p53											
Negative	772	76.5	76/732	10.4	<0.0005	108/718	15.0	0.264	27/687	3.9	0.120
Positive	237	23.5	51/230	22.2		40/220	18.2		14/217	6.5	
HER2											
Negative	774	84.9	107/756	14.2	0.058	132/740	17.8	<0.0005	34/709	5.7	0.764
Positive	138	15.1	11/135	9.3		7/133	5.3		5/120	4.2	
Age (years)											
≥50	668	62.0	65/603	10.8	0.006	80/580	13.8	0.033	19/570	3.3	0.045
<50	410	38.0	64/379	16.9		71/375	18.9		22/361	6.1	
											
*BRCA1*	46	4.3	6/34	17.6	0.250^b^	13/33	39.4	<0.0005^b^	1/43	2.3	0.866^b^
*BRCA2*	40	3.7	2/26	7.7	0.599^b^	7/26	26.9	0.011^b^	0/34	0	0.326^b^
Non-*BRCA1*/*BRCA2*	358	33.2	44/333	13.2	0.383^b^	67/324	20.7	<0.0005^b^	11/320	3.4	0.624^b^
Two affected	270	25.0	40/253	15.8	0.087^b^	30/244	12.3	0.469^b^	20/209	9.6	0.001^b^
Only sporadic	364	33.8	37/336	11.0	Ref.	34/328	10.4	Ref.	9/325	2.8	Ref.

^a ^Positive staining/samples available. ^b^Compared with 'only sporadic'. CK, cytokeratin; HER, human epidermal growth factor receptor.

*BRCA1 *associated cancers were more often high grade, ER negative, PR negative, p53 positive and HER2 negative, as well as CK 14 positive, as compared with all other groups (Table 2). CK-5/6 frequency was also higher but not significantly so if compared with non-*BRCA1*/*BRCA2 *or sporadic cancers. However, in multivariate analysis, including all factors from Table 2, neither CK-5/6 nor CK-14 were independent predictors of *BRCA1 *mutations. After stepwise removal of markers that were not significant at the 5% level, independent predictors were found to be age at onset, HER2 negativity and ER and/or PR status, depending on the analysis. In the analysis comparing *BRCA1 *tumours with sporadic tumours, the factors in the final model were ER (for negative status: odds ratio [OR] = 11.2, 95% confidence interval [CI] = 4.3 to 29.2), HER2 (for negative status: OR = 4607.4, 95% CI = 0 to 3.8 × 10^21^) and age at onset (for age <50 years: OR = 4.3, 95% CI = 1.6 to 11.2). Compared with non-*BRCA1*/*BRCA2 *families the final model included PR (for negative status: OR = 10.0, 95% CI = 3.5 to 28.6), HER2 (for negative status: OR = 4309.7, 95% CI = 0 to 3.3 × 10^24^) and age at onset (for age <50 years: OR = 5.0, 95% CI = 1.9 to 13.1).

**Table 2:** Association of clinicopathological features among tumours of *BRCA1 *families, *BRCA2 *families, non-*BRCA1*/*BRCA2 *families and two-affected families compared with tumours from patients with sporadic breast cancer

	*BRCA1 *cases			*BRCA2 *cases			Non-*BRCA1*/*BRCA2*			Two affected			Sporadic	
	
	*n*	%	*P*	*n*	%	*P*	*n*	%	*P*	*n*	%	*P*	*n*	%
All	46			40			358			270			364	
Oestrogen receptor														
Negative	33	75.0	<0.0005	15	38.5	0.013	79	24.3	0.294	64	24.9	0.248	75	20.9
Positive	11	25.0		24	61.5		246	75.7		193	75.1		283	79.1
Progesterone receptor														
Negative	37	84.1	<0.0005	21	53.8	0.014	115	35.6	0.657	93	36.3	0.548	122	34.0
Positive	7	15.9		18	46.2		208	64.4		163	63.7		237	66.0
Grade														
I	1	2.2	<0.0005	8	20.0	0.933	82	23.5	0.717	59	21.9	0.974	81	22.3
II	11	24.4		17	42.5		154	44.1		117	43.3		154	42.4
III	33	73.3		15	37.5		113	32.4		94	34.8		128	35.3
T														
1	21	46.7	0.079	18	54.5	0.516	229	65.6	0.144	162	60.4	0.976	219	60.3
2 to 4	24	53.3		15	45.5		120	34.4		106	39.6		144	39.7
N														
0	32	71.1	0.026	15	44.1	0.289	214	61.1	0.043	142	53.8	0.965	193	53.6
1	13	28.9		19	55.9		136	38.9		122	46.2		167	46.4
M														
0	41	93.2	0.570	28	90.3	0.245	338	98.8	0.005	255	96.6	0.386	335	95.2
1	3	6.8		3	9.7		4	1.2		9	3.4		17	4.8
p53														
Negative	25	62.5	0.017	26	76.5	0.707	261	76.5	0.399	193	75.1	0.233	267	79.2
Positive	15	37.5		8	23.5		80	23.5		64	24.9		70	20.8
HER2														
Negative	30	100	0.017	27	100	0.023	256	85.3	0.598	192	82.1	0.587	269	83.8
Positive	0	0		0	0		44	14.7		42	17.9		52	16.2
CK-5/6														
Negative	28	82.4	0.250	24	92.3	0.599	289	86.8	0.383	213	84.2	0.087	299	89.0
Positive	6	17.6		2	7.7		44	13.2		40	15.8		37	11.0
CK-14														
Negative	20	60.6	<0.0005	19	73.1	0.011	257	79.3	<0.0005	214	87.7	0.469	294	89.6
Positive	13	39.4		7	26.9		67	20.7		30	12.3		34	10.4
CK-17														
Negative	42	97.7	0.866	34	100	0.326	309	96.6	0.624	189	90.4	0.001	316	97.2
Positive	1	2.3		0	0		11	3.4		20	9.6		9	2.8
Age (years)														
≥50	16	34.8	<0.0005	22	55.0	0.004	215	60.1	0.035	97	35.9	0.356	246	67.6
<50	30	65.2		18	45.0		143	39.9		173	64.1		118	32.4

CK, cytokeratin; HER2, human epidermal growth factor receptor 2.

*BRCA2*-associated tumours were also significantly more often negative for ER, PR and HER2, and detected at earlier age than sporadic tumours or non-*BRCA1*/*BRCA2 *tumours. CK-14 was expressed more frequently than among sporadic cancers. However, in multivariate analysis, including all significant variables from univariate analysis, the only independent predictors of *BRCA2 *mutation were the age at onset under 50 years (OR = 3.7, 95% CI = 1.5 to 8.7), PR negativity (OR = 2.9, 95% CI = 1.2 to 6.9) and HER2 negativity (OR = 3705.2, 95% CI = 0 to 2.5 × 10^22^), as compared with sporadic tumours.

Tumours from familial non-*BRCA1*/*BRCA2 *patients were similar to sporadic ones, although they were of lower stage and CK-14 was expressed more often than among sporadic tumours (Table 2). Age at onset was slightly younger than among patients with sporadic breast cancer (median age at onset: 54 and 57 years; *P *= 0.001). Tumours from families with two affected members did not differ from sporadic tumours.

## Discussion

In this study expression of CK-5/6, CK-14 and CK-17 was found to be strongly associated with the subgroup of tumours negative for ER and HER2, as suggested previously for the basal subgroup of breast tumours [3]. However, although our study confirms that basal CKs, especially CK-14, can help to identify *BRCA1 *mutation carriers, this effect was weaker than in previous studies.

Because of differences in antibodies, staining and tissue processing procedures in different studies, it is difficult to compare findings between studies directly. For example, we chose a cut-off point (>10%) for CKs that results in clearly positive staining. Previously, rather different cut-off points had been used, from any positivity to 20%. However, the frequencies in the present study among unselected breast cancer patients correlate with the previous literature, in which about 2% to 19% of unselected breast cancers were positive for CK-5, CK-6 and CK-14 [7-9,24,25]. Moreover, the subgroups in our study are comparable because the material was collected uniformly from the same hospital, and microarray tissue blocks enable staining of all samples at the same time and under the same conditions. Highly significant correlations between findings obtained using this kind of multicore system and of whole sections of the original blocks have also been demonstrated [26,27].

Similarly to previous studies, we found that expression of any one or all of the markers CK-5/6, CK-14 and CK-17 correlates with ER negativity [4,8,24], PR negativity [8], high grade [4,8,24,25], early age at diagnosis [4,24,25] and p53 positivity [4]. Numerous studies have reported that basal CKs are more frequently positive in *BRCA1 *tumours than in sporadic breast cancers [6-9]. In our study as well, CK-14 was significantly associated with *BRCA1 *carrier status, and CK-5/6 frequency was also higher than among sporadic breast cancers but the difference was not statistically significant. In the study conducted Lakhani and coworkers [9], 57.6% of the *BRCA1*-associated tumours stained positive for CK-5/6 and 60.6% for CK-14. Corresponding figures in our study were 17.6% and 39.4%, although the percentages in control populations in both studies were similar (Table 2).

In the study conducted by Foulkes and colleagues [6], CK-5/6 was detected in 56% of the 72 ER/HER2-negative cancers, and the frequency was even higher (88%) among the 17 *BRCA1 *mutation carriers with ER/HER2-negative cancers. In our study CK-5/6 positivity was detected in a large proportion (33%) of the 167 ER/HER2-negative cancers as well, but only in 25% of the 20 ER/HER2-negative and *BRCA1*-related cancers. CK-14 was detected in 35% of 161 ER/HER2-negative cancers and 37% of the ER/HER2-negative and *BRCA1*-positive group.

Few studies have used multivariate analysis to address the significance of basal CKs in predicting *BRCA1 *mutations. In the study conducted by Lakhani and colleagues [9], CK-14, CK-5/6, CK-17, osteonectin, epidermal growth factor receptor, ER and grade were included in a multiple regression model. They found that independent markers for *BRCA1 *positivity were ER, CK-5/6 and CK-14. In our multivariate analysis, including all factors significant in univariate analysis, the studied basal CKs were not independent predictors of *BRCA1 *mutation status. However, in the multivariate analysis with only basal CKs together with ER and grade, as in the study by Lakhani and colleagues [9], the remaining significant factors were CK-14 (OR = 3.3, 95% CI = 1.4 to 7.9) and ER (OR = 6.2, 95% CI = 2.7 to 14.2). Thus, the effect of CK-14 was similar to that detected by Lakhani and colleagues. However, the CK-5/6 result was different from their study, because it was not significant in univariate or multivariate analysis. One factor that may affect this is the strong association of CK-5/6 with age at diagnosis, as shown in Table 1. The tumours included in our study were unselected for age, and about 35% of the *BRCA1 *tumours in our study were diagnosed at age 50 years or older. We previously showed that tumours of *BRCA1 *and *BRCA2 *carriers aged 50 years or older differ significantly from those of younger patients [28]. However, CK-14 expression correlates strongly with the HER2 status, as well as with ER and PR status, and if we included all of these studied variables in the logistic regression analysis, then the remaining independent factors for *BRCA1 *mutation, in the final model, were only age at onset, HER2 negativity and ER and/or PR status, depending on the analysis. Age at onset, HER2 and PR were not included in multivariate analysis conducted by Lakhani and colleagues [9].

We also detected CK-14-positive staining among 27% of *BRCA2 *associated tumours and 21% of the non-*BRCA1*/*BRCA2 *associated tumours. These proportions are significantly higher than the 10% among the sporadic tumours (*P *= 0.011 and *P *< 0.0005, respectively). This finding could be influenced by chance in a low population frequency with multiple testing, or by the younger age in these subgroups compared with the older patients with sporadic breast cancer, because CK expression also correlates with tumours occurring at earlier ages. In multivariate analysis, CKs were not independently associated with BRCA2 mutation status. To exclude the possibility of missed *BRCA1 *mutations among non-*BRCA1*/*BRCA2 *families, we conducted the same analysis after excluding families with ovarian cancer cases, but this did not alter the results. In the study conducted by Lakhani and colleagues [9], none of the basal markers was significantly associated with *BRCA2 *status. However, in their study as well, the frequencies of CK-5/6 (15%) and CK-14 (24%) were higher among *BRCA2 *cases than among controls (7% and 12%).

## Conclusion

ER and PR strongly correlate with each other (Pearson's R = 0.657, *P *< 0.0005 in this study), and both of them were found to be effective in predicting *BRCA1 *mutation. However, although our study confirms that basal CKs can help to identify *BRCA1 *mutation carriers, this effect was weaker than previously suggested and CKs did not independently predict *BRCA1 *mutation either in sporadic or familial breast cancer cases. One factor that may affect this is the strong association of CK-5/6 with age at diagnosis, because the tumours included in our study were unselected for age and about 35% of the *BRCA1 *tumours in our study were diagnosed at age 50 years or older. In our material CKs also correlated strongly with many other tumour characteristics, such as ER negativity, PR negativity, high grade, p53 positivity, HER2 negativity and age at onset. If we consider all factors analyzed, the most effective independent predictors of *BRCA1 *mutations were age at onset, HER2 status and either ER or PR status. The same factors appear also to predict *BRCA2 *mutations, although not as strongly as *BRCA1 *mutations.

## Abbreviations

CI = confidence interval; CK = cytokeratin; ER = oestrogen receptor; HER = human epidermal growth factor receptor; OR = odds ratio; PR = progesterone receptor; TMA = tissue microarray.

## Competing interests

The authors declare that they have no competing interests.

## Authors' contributions

All authors were involved in critically revising the manuscript and gave their approval for the manuscript to be published. Furthermore, HE participated in patient and tumour sample collection, performed statistical analysis and drafted the manuscript. MH and AR scored and interpreted the immunoassays. PH studied the original tumour samples for histological diagnosis and grading, and organized immunoassays. OK participated in designing and coordination of the study and helped to draft the manuscript. AT carried out molecular genetic studies. KA participated in patient collection and was responsible for genetic counselling of patients. CB participated in organizing patient and tumour collections. HN participated in the study design and coordination, and helped to draft the manuscript.
